# Novel 3D Force Sensors for a Cost-Effective 3D Force Plate for Biomechanical Analysis

**DOI:** 10.3390/s23094437

**Published:** 2023-05-02

**Authors:** Jonathan D. Miller, Dimitrije Cabarkapa, Andrew J. Miller, Lance L. Frazer, Tylan N. Templin, Travis D. Eliason, Samuel K. Garretson, Andrew C. Fry, Cory J. Berkland

**Affiliations:** 1 Higuchi Biosciences Center, University of Kansas, Lawrence, KS 66045, USA; 2 Axioforce LLC, St. Louis, MO 63141, USA; 3 Health, Sport, and Exercise Sciences, University of Kansas, Lawrence, KS 66045, USA; 4 Southwest Research Institute, San Antonio, TX 78238, USA; 5 Department of Chemical and Petroleum Engineering, University of Kansas, Lawrence, KS 66045, USA; 6 Department of Pharmaceutical Chemistry, University of Kansas, Lawrence, KS 66045, USA

**Keywords:** force plate, biomechanics ground reaction force, magnetic materials, composite materials, sensors

## Abstract

Three-dimensional force plates are important tools for biomechanics discovery and sports performance practice. However, currently, available 3D force plates lack portability and are often cost-prohibitive. To address this, a recently discovered 3D force sensor technology was used in the fabrication of a prototype force plate. Thirteen participants performed bodyweight and weighted lunges and squats on the prototype force plate and a standard 3D force plate positioned in series to compare forces measured by both force plates and validate the technology. For the lunges, there was excellent agreement between the experimental force plate and the standard force plate in the X-, Y-, and Z-axes (r = 0.950–0.999, *p* < 0.001). For the squats, there was excellent agreement between the force plates in the Z-axis (r = 0.996, *p* < 0.001). Across axes and movements, root mean square error (RMSE) ranged from 1.17% to 5.36% between force plates. Although the current prototype force plate is limited in sampling rate, the low RMSEs and extremely high agreement in peak forces provide confidence the novel force sensors have utility in constructing cost-effective and versatile use-case 3D force plates.

## 1. Introduction

Measuring kinetics from human movements is an important field of discovery in biomechanics research and a growing practice in sports performance [1,2]. Potentially the most important and sought-after tool in this space is the 3D force plate. Force plates find their value in biomechanics research and sports performance due to their ability to collect important information about an athlete’s power, fatigue, and stretch-shortening cycle capabilities from simple tests that require minimal setup time and familiarization on the part of the athlete [2,3,4,5,6]. For example, Read et al. [7] performed a battery of tests to investigate potential kinetic and kinematic risk factors for injury in young elite football players. The factor with the greatest impact on injury risk was high single-leg counter movement jump ground reaction force asymmetries between legs, indicating the utility and sensitivity of kinetic factors measured by force plates.

However, currently available 3D force plates lack portability, are not suitable for outdoor use, and are expensive, often costing upwards of USD 10,000 to USD 20,000 [8]; most research and sports programs are limited to a single force plate or a small number of force plates. Indeed, many smaller research and sports programs are inhibited from purchasing a single 3D force plate and may opt for a cheaper unidimensional or two-dimensional force plate, which lacks critical information needed for many biomechanical analyses. As a response to this lack of affordability for high-quality force plates, several researchers have set out to design, construct, and test force plate systems that can be replicated at much lower costs [8,9,10,11,12]. However, of these recent attempts, only two experimental force plates measured forces in three dimensions, and both were limited to low force capacities, inhibiting use for sports performance [8,9]. For example, Ferryanto et al. used recently developed affordable 3D load cells [13] to construct a 3D force plate with reasonable accuracy. However, the total cost of raw materials still amounted to USD 1400, and the force plate was limited to a maximum load of just 1500 N, which is insufficient for vertical jump analysis or other sport performance applications. The primary driver of the high cost of 3D force plates is the complex manufacturing of the 3D load cells [13]. Thus, while these efforts have added significantly to the literature, it remains necessary to develop a low-cost 3D force plate that has sufficient capacity for a wide variety of use cases.

Design methods for magnetic soft 3D force sensors have recently been described [14] and may provide a platform for cost-effective 3D force measurement systems. Therefore, we present the following work as a potential option to alleviate the cost-inhibition and narrow use-case options of the current 3D force plate market by designing and validating a force plate prototype that leverages these recently discovered 3D force sensors, which cost less than USD 25 each.

A force plate prototype was constructed using aluminum plates and four of the novel magnetic 3D force sensors. The experimental force plate was positioned atop a standard Bertec 3D force plate, which served as ground truth, and 3D forces from dynamic human movements were recorded and analyzed to calibrate and validate the prototype force plate. Based on data presented in our previous work [14] indicating the high accuracy of the sensors during controlled studies, we hypothesized the experimental force plate would show a high level of agreement with the standard force plate after calibration. However, the low sampling rate of the current prototype (100 Hz) would leave room for improvement, and thus further iteration is desired.

## 2. Materials and Methods

### 2.1. Three-Dimensional Force Sensor Design and Fabrication

Magnetic soft 3D force sensors were constructed according to methods presented in detail by Miller et al. [14]. All sensors were 10 mm diameter by 3 mm height cylinders composed of PDMS silicone rubber elastomer (Sylgard 184, 10:1 base to curing agent ratio, Dow Chemical Company; Pevely, MO, USA), with a discreet cylindrical portion of the construct composed of a silicone-magnetic powder composite (80% magnetic powder by mass). The magnetic powder used in the composite was a neodymium iron boron alloy with an average particle size of <10 µm (American Elements, Los Angeles, CA, USA). The geometry of this magnetic element was a 2 mm diameter, 2.25 mm tall cylinder. Once the silicone constructs were completely cured, the dipoles of the magnetic particles were aligned according to methods described by Miller et al. [14].

The silicone construct was adhered to an MLX90393 magnetometer by a silicone-based adhesive with the magnetic element positioned away from the magnetometer, such that a 0.75 mm “gap” of pure silicone rubber existed between the magnetic element and the magnetometer. A detailed description and illustration of the fabrication and design of the sensors are presented by Miller et al. [14].

### 2.2. Force Plate Fabrication

**Figure 1:**
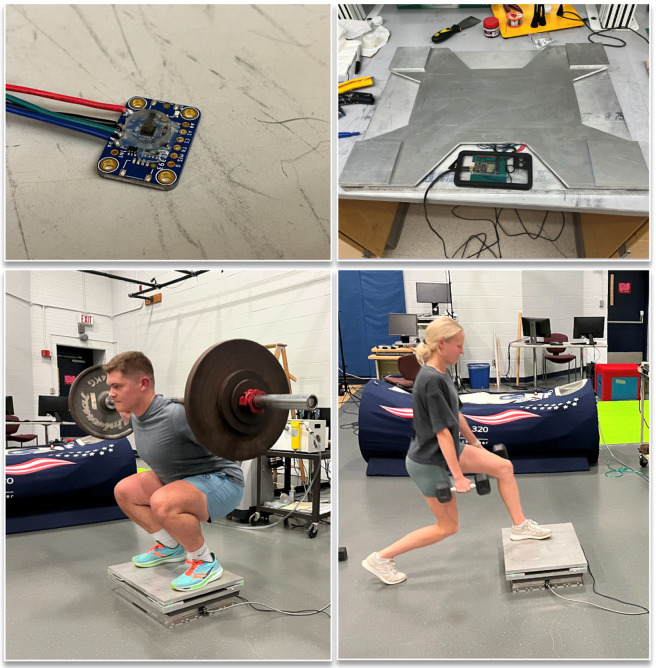
Images of a single sensor unit (**top left**), the assembled experimental force plate (**top right**), and participants performing weighted squats (**bottom left**) and lunges (**bottom right**) atop the Bertec (ground truth) force plate and the experimental force plate.

A force plate was fabricated by adhering one of the novel 3D force sensors described herein to each of the four corners of an aluminum plate (Figure 1). First, the sensors were encased in a 40 × 40 × 5.5 mm pad of pure silicone to increase the physical area of the sensors, which was adhered to the aluminum plates, thereby decreasing the pressure that they would experience and controlling the amount of deformation to less than 0.5 mm during testing of human subjects. This ensures participants would not perceive the deformation of the sensors when performing movements on the force plate. These constructs then adhered to each corner of a 600 × 450 mm, 19 mm thick 6061-T651 aluminum plate (Midwest Steel Supply, Inc, Rogers, MN) which was sufficiently rigid for use as a force plate for biomechanical analysis. [8] The material properties of the plate are as follows:

Young’s Modulus of Elasticity: 68.9 GPa;
Bearing Yield Strength: 386 MPa;
Flexural Modulus: 299 MPa.

The bottom of each sensor was also adhered to a thinner aluminum plate, as is standard in force plate fabrication, to ensure forces were properly transmitted through the sensors. The sensors were wired to a microcontroller, which transmitted the data via USB to a nearby computer at 100 Hz. Custom software was written for live visualization, recording, and storing of the 3D ground reaction force data collected by the experimental 3D force plate. The total cost for the components within the force plate was less than USD 300.

### 2.3. Experimental Methods

Nine males and four females (height = 178.8 ± 11.2 cm; body mass = 81.8 ± 17.3 kg; age = 28.4 ± 7.1 years) volunteered to participate in this study. All participants were recreationally active (endurance, resistance, or mixed training > 2×/week) at the time of the study and did not report any current or previous musculoskeletal injuries that could inhibit their performance of the tasks required for the study. All testing procedures performed in this study were previously approved by the university’s Institutional Review Board (STUDY00148420), and all participants provided written informed consent.

Participants were asked to estimate their squat 1-repetition maximum (1-RM) to standardize efforts on weighted squat and lunge movements which would be performed on the force plates. All participants performed a 5-min treadmill warm-up and were also allowed time to perform any additional dynamic warm-up exercises desired. Following the warm-up, participants performed several sets of bodyweight and weighted squats, as well as lunges on the force plates. The data from these movements were used for analysis to compare the experimental force plate against a standard Bertec (Columbus, OH, USA) model 4060-08 3D force plate.

For the squats, participants performed a set of 5 repetitions at bodyweight, 20% of their estimated squat 1-RM, and 40% of their estimated squat 1-RM using a barbell and free weights for resistance. For the lunges, participants performed a set of 8 repetitions (4 each leg) at bodyweight; 10% estimated squat 1-RM, and 20% estimated squat 1-RM using dumbbells for weights. To ensure the experimental force plate experienced significant forces in each direction of each axis, lunges were performed in each horizontal direction on the force plate each set. That is, for each set of 8 lunges, 2 lunges (1 each leg) were performed on each side of the force plate, such that the participant moved around the force plate during the set, rotating sides of the force plate after every two lunges.

For all tests, the Bertec force plate was positioned on the ground, and the experimental force plate was positioned on top of the Bertec force plate with a separate rigid aluminum plate between them to ensure all forces experienced by the prototype force plate were properly transferred through the Bertec force plate (See Figure 1). A weighted ball was dropped on the force plates at the beginning of each recording in order to sync the data from the Bertec and the experimental force plate. A comparison of some of the relevant characteristics of each force plate can be seen in Table 1.

### 2.4. Calibration

The Bertec force plate was calibrated using standard 2-point calibration procedures for each axis. However, due to the nature of the novel 3D force sensors used for the experimental force plate in the current study, a standard 2-point calibration is insufficient for properly calibrating the force plate in 3 dimensions. The magnetic sensors used for this force plate have a nonlinear relationship with force [14], and the 3 axes require deconvolution. Therefore, an optimization procedure was employed to calibrate the prototype force plate to the standard force plate as follows.

X, Y, and Z data from the squat and lunge movements from the lightest and heaviest subject from both force plates were time synced, and a vector of data was created for the prototype force plate defined by the following:

DATA_prototype_ = [X_1m_1_, X_2m_1_, X_3m_1_, …, X_tm_1_, Y_1m_1_, Y_2m_1_, Y_3m_1_, …, Y_tm_1_, Z_1m_1_, Z_2m_1_, Z_3m_1_, …, Z_tm_1_, X_1m_2_, …]

where X, Y, and Z denote the raw data trace for each of the coordinate directions, the first subscript denotes the time point, and m_i denotes the movement (squat, lunge). Similarly, a corresponding vector was made from the standard force plate data. For each coordinate direction and for each sensor, a power law equation is assumed to convert raw sensor data to a force (4 sensors × 3 coordinate directions = 12 unique equations):

Force_j_ = B·Raw_j_ + C^e^·Raw_j_

where j denotes each time point, and B, C, and e are unknown parameters. Thus, a total of 36 parameters were to be found via optimization. The DATA_prototype_ vector was converted to a force vector using the conversion equations. A trust-region method for nonlinear least squares optimization was performed using DAKOTA (Sandia National Labs, Albuquerque, NM, USA) to find the unknown parameters that minimize the root mean square error between the force vector predicted from the prototype force plate and the standard force plate force vector. With an optimized calibration, the resultant force for each coordinate direction is then calculated by passing the raw force sensor signals through the Force_j_ equations above. Thus, the squat and lunge data from the heaviest and lightest subject (in terms of body weight) were used only for calibrating the experimental force plate and were excluded from any further analysis.

### 2.5. Statistical Analysis

Data from the Bertec force plate was down-sampled to 100 Hz according to the sampling rate of the experimental force plate. Root mean squared errors (RMSE) were calculated on the entire data trace from each set of lunges and squats for each participant. Peak forces from each axis (X, Y, and Z) were determined for both force plates from lunges, and peak forces from the Z-axis only were determined for both force plates from squats. Horizontal peak forces (X-axis and Y-axis) from squat movements were excluded from analysis as many repetitions had no easily discernable peak in the horizontal directions. The peak forces were compared across force plates by calculating two-way, random effect, single measures, absolute agreement, intraclass correlation coefficients (ICC), [15] as well as mean biases performed on the same peaks. For these statistical models, data were collapsed across weight for lunges (bodyweight, 10% squat 1-RM, 20% squat 1-RM) and for squats (bodyweight, 20% squat 1-RM, 40% squat 1-RM) but were analyzed separately for each axis (X, Y, and Z) and movement type (lunge and squat).

## 3. Results

**Figure 2:**
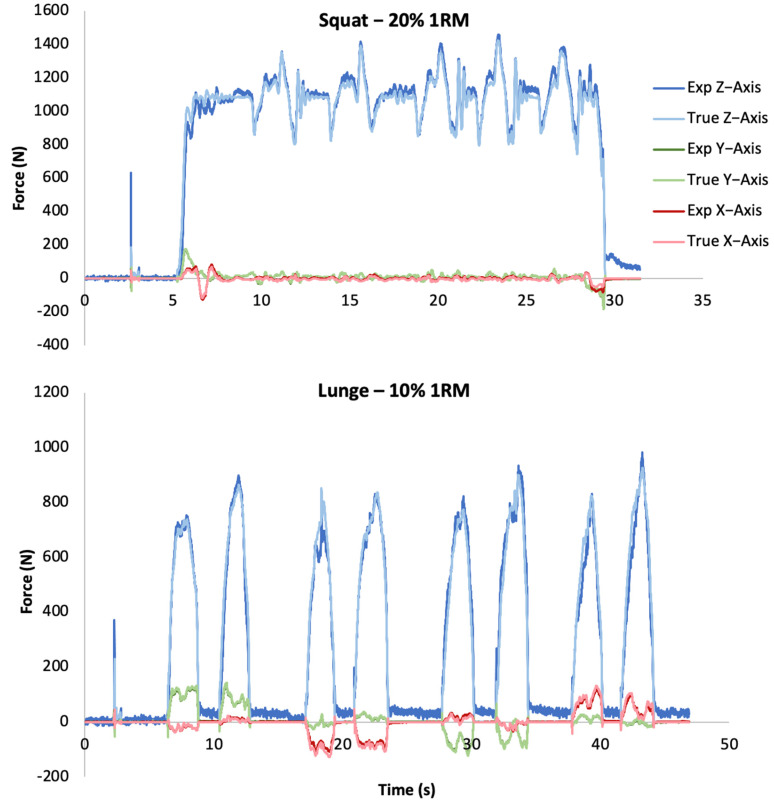
Force vs. time plotted for each axis of the experimental force plate and the Bertec force plate (ground truth) for a representative set of squats (**top**) and lunges (**bottom**).

The experimental force plate showed relatively low RMSE and minimal mean bias in comparison to the Bertec force plate, and there was excellent agreement for peak forces between the force plates (Table 2). The experimental force plate examined in this study would require an increased sampling rate to improve the accuracy of the assessment of human biomechanics. However, this work serves as a proof-of-concept for the use of the silicone-magnetic composite sensors developed by Miller et al. [14] within force plates for analyzing human kinetics. Examples of 3D forces plotted for both force plates from the same movement can be seen in Figure 2.

### 3.1. Absolute and Relative RMSE

The absolute and relative RMSE for each axis of both movements is displayed in Table 2. The relative RMSE values indicate the experimental force plate performed with lower relative error in the shear (X and Y) axes than the vertical (Z) axis during both lunges and squats. The vertical axis during lunges displayed the highest error.

### 3.2. Peak Force ICCs and Mean Biases

**Figure 3:**
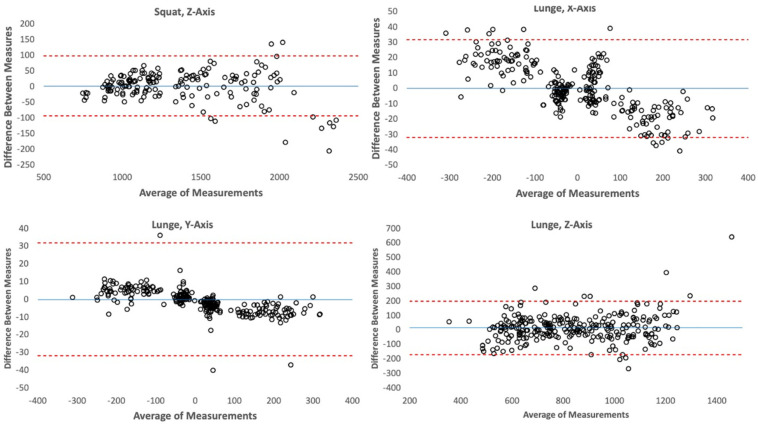
Bland–Altman plots for the peak forces measured during each repetition from all participants for the X-, Y-, and Z-Axes of the Lunge movements as well as the Z-Axis for the squat movements. Residuals and averages of measures are in units of Newtons (N). Blue line represents the mean, and red dotted lines represent 95% confidence intervals.

For the lunges, there was excellent agreement between the experimental force plate and the Bertec force plate in the X-axis (r = 0.996, *p* < 0.001), Y-Axis (r = 0.999, *p* < 0.001), and Z-axis (r = 0.950, *p* < 0.001). For the squats, there was excellent agreement between the experimental force plate and the Bertec force plate on the Z-axis (r = 0.996, *p* < 0.001). Agreement between the force plates in terms of peak forces in the X-axis and Y-axis was not assessed as a lack of major horizontal forces during the squats led to some recordings having no discernable peaks in these axes. Bland–Altman plots of the peak forces for the Z-axis from squat movements and from each axis from the lunge movements are presented in Figure 3.

Mean biases for the X-, Y-, and Z-Axes of the lunges, as well as for the Z-Axis of the squats, are displayed in Table 1. Mean biases were quite minimal (<2 N) except for peak forces measured in the Z-Axis from the lunges (~15 N), indicating the prototype force plate was typically unbiased except that it tended to overestimate force in the Z-axis during lunges.

## 4. Discussion

The experimental force plate fabricated using recently discovered silicone-magnetic powder composite sensors performed well in comparison to a standard Bertec 3D force plate. This provides a promising platform for building 3D force plates for the assessment of human kinetics that is not as cost prohibitive or limited as current standard 3D force plate technology is. The force plate described herein cost significantly less than previous attempts at low-cost 3D force plates in the literature [8,9] while having much greater load capacity, enabling it to be used in sport performance applications. In general, the relative RMSEs between the experimental force plate and the standard force plate was ~3% or less, except in the case of the Z-axis during the lunges. Most importantly, the experimental force plate showed extremely high agreement with the standard force plate in terms of the ICCs (0.950–0.999) of the peak forces, suggesting highly consistent measurement.

One limitation of this study is the low sampling rate (100 Hz) of the experimental force plate studied. Previous research has indicated low sampling rates bias kinetic measurements and lead to inaccuracies [16]. At least 1000 Hz is the recommended minimum sampling rate for quantifying kinetic factors of human movements. Future iterations of force plates built with this technology should have significantly increased sampling rates, as 1000–2000 Hz is the standard for human kinetic assessment. It is likely that much of the error observed in the experimental force plate in each axis that was present (~1–5%) is due to this low sampling rate. According to the Bland–Altman plots in Figure 3. The X- and Y-axis offsets for the prototype force plate are biased towards lower magnitude responses to force in comparison to the Bertec. It is likely this bias is also due to the lower sampling rate of the prototype force plate, which will prevent it from adequately capturing higher frequency components of the force signal, such as these force peaks. In addition, another limitation is that a controlled 3D calibration procedure for each of the individual sensors used in the force plate has not yet been developed. Therefore, the current prototype must implement a calibration procedure that considers all the sensors in tandem and cannot discern what the true calibration parameters should be for each individual sensor. This results in a limited ability to control for fabrication differences leading to different sensitivities between each sensor. It is likely that force plates built with this sensor technology that addresses these limitations would have significantly reduced error in comparison to standard force plate technology.

In addition to higher sampling rates and a procedure for individual sensor calibration, future work exploring subsequent versions of this force plate could be built with lighter weight yet still rigid materials such as aluminum honeycomb plates, enabling the force plate to be more portable. This portability is an especially important opportunity because the sensors used are natively waterproof, as they are composed of silicone. This could allow for a cost-effect, portable force plate that could be used outdoors, significantly increasing the use cases of 3D force plates.

### Considerations for Wearable Technology Implementations

The silicone-magnetic sensors used in the experimental force plate within this study are made of biocompatible and soft yet mechanically robust silicones. This enables the potential to use this 3D force sensor platform in wearable technologies such as shoe insoles and helmets [14] as well as in other medical technologies. This study provides an initial validation of these magnetic silicone composite sensors for analyzing 3D ground reaction forces from human movements within a force plate. However, because of the potential for this technology within wearables, future studies should validate these sensors in different wearable technologies against gold-standard 3D force measurement devices.

## 5. Conclusions

Here, we have presented a prototype solution for a non-cost-prohibitive 3D force plate. The experimental force plate was constructed with a recently developed 3D sensor technology that leverages a silicone-magnetic composite material. The force plate was tested against a standard 3D force plate during lunge and squat movements performed at bodyweight and in weighted conditions. The analysis indicated low RMSE (1.17–5.36%), mean biases (0.01–14.56 N), and high peak force agreement (ICC: 0.950–0.999) between force plates indicating its accuracy and reliability. In addition, the force plate was significantly cheaper with drastically increased load capacity in comparison to previous attempts at cost-effective 3D force plates in the literature, [8,9] supporting its utility for assessment of sports performance-related human kinetics (e.g., vertical jump testing). However, future iterations of the force plate with higher sampling rates and individual sensor calibration procedures are needed for improved accuracy.

## Figures and Tables

**Table 1:** Comparison of dimensions and data parameters between the prototype force plate and the Bertec force plate.

	Force Plate	
	Bertec 4060-80	Prototype
length (mm)	600	600
width (mm)	400	450
height (mm)	83	40
sample frequency (Hz)	2000	100
maximum load (N)	10,000	14,000 *
resolution (N/lsb)	0.19	See Miller et al. [14]

* Estimated based on mechanical testing of individual sensors and mechanical properties of aluminum plates.

**Table 2:** Mean ± standard deviations of the absolute values of the peak forces for the lunges and the squats. Mean Biases of the peak forces. Absolute and relative (to the range of the data trace) RMSE for the entire data traces for lunges and squats.

		Lunges			Squats	
	X-Axis	Y-Axis	Z-Axis	X-Axis	Y-Axis	Z-Axis
Bertec (Ground Truth)	112 ± 85	110 ± 82	825 ± 213	-	-	1368 ± 397
Experimental Force plate	104 ± 75	105 ± 81	843 ± 225	-	-	1370 ± 388
Mean Bias (N)	0.01	−1.18	14.56	-	-	1.95
Absolute RMSE	7.27	4.29	53.69	6.56	3.56	51.14
Relative RMSE	2.09	1.17	5.36	2.51	1.62	3.66

## Data Availability

Data available upon request.
